# Therapist perceptions of a rehabilitation research study in the intensive care unit: a trinational survey assessing barriers and facilitators to implementing the CYCLE pilot randomized clinical trial

**DOI:** 10.1186/s40814-019-0509-3

**Published:** 2019-11-12

**Authors:** Julie C. Reid, Devin S. McCaskell, Michelle E. Kho

**Affiliations:** 1 Faculty of Health Sciences, School of Rehabilitation Science, Institute of Applied Health Sciences, McMaster University, Room 403, 1400 Main Street West, Hamilton, ON L8S 1C7 Canada; 2 Department of Physiotherapy, St. Joseph’s Healthcare Hamilton, 50 Charlton Avenue East, Hamilton, ON L8N 4A6 Canada

**Keywords:** Questionnaire, Knowledge translation, Rehabilitation, Physical therapists

## Abstract

**Background:**

Rehabilitation interventions, including novel technologies such as in-bed cycling, could reduce critical illness-associated morbidity. Frontline intensive care unit (ICU) therapists often implement these interventions; however, little is known about their perceptions of engaging in clinical research evaluating these technologies.

**Objective:**

To understand frontline therapist perceptions of barriers and facilitators to implementing a pilot randomized controlled trial (RCT) of early in-bed cycling with mechanically ventilated patients in the ICU and outcome measures (CYCLE Pilot RCT; NCT02377830).

**Methods:**

We developed a 115-item, self-administered, electronic survey informed by 2 complementary knowledge translation (KT) models: the Capability-Opportunity-Motivation-Behaviour (COM-B) system and the Theoretical Domains Framework (TDF). We included demographics and 3 sections: Rehabilitation Practice and Research, Cycling, and Physical Outcome Measures. Each section contained items related to the COM-B system and TDF domains. Item formats included 7-point Likert-type scale questions (1 = strongly disagree, 7 = strongly agree) and free-text responses. We invited therapists (physiotherapists, occupational therapists, and therapy assistants) who participated in the international, multi-center, CYCLE Pilot RCT to complete this cross-sectional survey. We descriptively analyzed results by survey section, COM-B attribute, TDF domain, and individual question within and across sections. We identified barriers based on items with median scores < 4/7.

**Results:**

Our response rate was 85% (45/53). Respondents were from Canada (67%), the USA (21%), and Australia (11%). The majority had a physiotherapy background (87%) and previous research experience (87%). By section, Rehabilitation Practice and Research (85%; 95% confidence interval (CI) [82%, 87%]) was higher than Cycling (77%; 95% CI [73%, 80%]) and Outcome Measures (78%; 95% CI [75%, 82%]). Across the 3 sections, Motivation was lower than Capability and Opportunity. The most common Motivation barrier was the emotion TDF domain, related to the time required to conduct cycling and outcome measures (median [1st, 3rd quartiles] 3/7 [2, 6]).

**Conclusions:**

Frontline ICU therapists had positive perceptions of research engagement. However, we identified barriers related to Motivation, and concerns regarding time to implement the research protocol. Our results can inform specific KT strategies to engage frontline ICU therapists and optimize protocol implementation in critical care rehabilitation research.

## Introduction

With advancements in medical technology, more people are surviving critical illness [1]. However, survivors of an intensive care unit (ICU) stay often experience substantial physical disability [2, 3]. Because of this disability, there has been a great focus on minimizing critical illness-associated morbidity through rehabilitation interventions started early in ICU [4, 5]. While frontline ICU therapists are often responsible for providing these interventions, we have limited knowledge of their perceptions of implementation as part of a research study.

Implementing interventions into clinical practice is challenging [6]. Complex interventions, such as physical rehabilitation, can be even more difficult to implement. For ICU patients, implementation is more complex due to the critical care environment, patients’ illness severity, and their receipt of life support therapies [7, 8]. For example, observational studies reported rehabilitation with mechanically ventilated (MV) ICU patients was uncommon [9, 10]. In a randomized controlled trial (RCT) of intensive versus routine physiotherapy in the ICU, the intervention group only received 23 min out of a planned 90 min of therapy per day [11]. Since intervention implementation is dependent on healthcare practitioner actions, understanding determinants of their behaviors are crucial [12, 13]. Knowledge translation (KT) is a field dedicated to the study of methods to move evidence into practice [14–16]. KT theory can help identify factors limiting implementation and develop strategies to overcome barriers and optimize facilitators [14, 17].

CYCLE (*C*ritical Care C*yc*ling to Improve *L*ower *E*xtremity Strength) is a multiphase, multidisciplinary, international research program evaluating the effectiveness of early in-bed cycling to improve functional outcomes for MV patients in ICU [18]. The CYCLE Pilot RCT occurred in seven ICUs and demonstrated that an RCT of cycling within the first 4 days of MV was feasible [18]. However, we identified potential challenges for future RCT, including frontline therapist ability to contribute to research. Therapist roles in implementing CYCLE included conduct of in-bed cycling with critically ill MV patients in the ICU, administration of physical function outcome measures, and data collection during cycling sessions and outcomes assessments. In advance of the full CYCLE RCT, we developed a self-administered survey to address the following research question, “What are CYCLE ICU therapists’ perceptions regarding conduct of clinical research, including barriers and facilitators to conducting early in-bed cycling with MV patients in the ICU and outcome measures?”

## Methods

### Survey development

We used two complementary and interrelated frameworks of behavior change to develop our survey. The Capability-Opportunity-Motivation-Behaviour system (COM-B) acknowledges the necessary interaction of capability, opportunity, and motivation attributes for behavior to occur [19]. We then used the Theoretical Domains Framework (TDF) [20, 21] to guide development of specific questions. The TDF is a synthesis of 33 theories of behavior change organized into 14 discrete domains [20]. Focused on healthcare practitioners, it is applied to KT strategies in healthcare [17]. Each TDF domain links to one of Capability, Opportunity, or Motivation in the COM-B framework and helps identify factors that could influence healthcare practitioners’ behaviors [21]. We used rigorous survey development and testing methods throughout [22] and followed the Checklist for Reporting Results of Internet E-Surveys [23].

#### Item generation, reduction, and question and answer stems

We reviewed a process evaluation of complex rehabilitation intervention implementation [24] and conducted focus groups with therapists experienced with in-bed cycling from TryCYCLE (the safety and feasibility phase of CYCLE [25]) to identify items for attitudes towards research, conduct of cycling, and outcome measures. We continued item generation until each TDF domain was represented at least once in the survey and until all sections contained items pertaining to Capability, Opportunity, and Motivation. We reduced items by identifying redundant questions and questions of limited relevance. Most questions were answered using a 7-point Likert-type scale (1 = strongly disagree, 7 = strongly agree). Further detail is found in Additional file 1.

#### Formatting

We used LimeSurvey (version 3.15.6+190108, Hamburg, Germany: LimeSurvey GmbH), a web-based, secure, ethics-compliant platform, which assigned participants a unique identifier to prevent duplicate survey completion.

### Testing

We conducted rigorous testing of the survey, including pre-, pilot, and clinical sensibility testing to evaluate feasibility, ease of administration, and face validity [22] (Additional file 1: Table S2). We revised the survey based on feedback. Once in its penultimate version, we reviewed survey questions to ensure representation of all TDF domains. Our final survey included participant demographics, three main sections (Rehabilitation Practice and Research, Cycling, Outcome Measures), and a free-text section for a total of 115 questions in five electronic pages (Additional file 2). Our pilot testing suggested 20–25 min for survey completion.

### Administration

We invited all ICU therapists (physiotherapists (PT), occupational therapists (OT), and therapy assistants) who received training for the CYCLE Pilot RCT and CYCLE Vanguard (additional internal pilot) studies to participate. We excluded those who did not participate in the studies (NCT02377830) [18]. Survey completion was voluntary and responses anonymous. We obtained consent electronically using a checkbox on survey initiation. Additional file 1 provides further administration details. The Hamilton Integrated Research Ethics Board approved this study.

### Statistical analysis

We calculated response rate as the proportion of returned surveys divided by the number of eligible respondents [26]. We included any returned questionnaire with at least one completed section. For the Cycling and Outcome Measure sections, we only included respondents who reported performing those roles. Further information about missing data is in Additional file 1.

We aggregated all Likert-type questions in two ways: (1) by individual section and (2) across the three sections. By section, we calculated the following: (a) sum score; (b) sum of Capability, Opportunity, and Motivation items; and (c) sum of items contributing to each TDF domain. For negatively framed questions (e.g., I felt overwhelmed by cycling with critically ill patients), we reversed the scale responses so all questions had the same polarity. We also examined data by item. If median item scores were < 4/7 or > 4/7, we classified those items as barriers and facilitators, respectively. We used free-text responses to contextualize the results of the Likert-type scale questions. Across the three sections, we summed items contributing to Capability, Opportunity, and Motivation.

We calculated descriptive statistics of continuous variables such as means and standard deviations or medians and 1st and 3rd quartiles if data were skewed. For categorical variables, we calculated counts and percentages. We completed visual inspection of descriptive data using boxplots and histograms and assessed normality using the Shapiro-Wilk test. We conducted analyses using Stata (version 14.2, College Station, Texas: StataCorp LP).

## Results

### Respondents

**Fig. 1:**
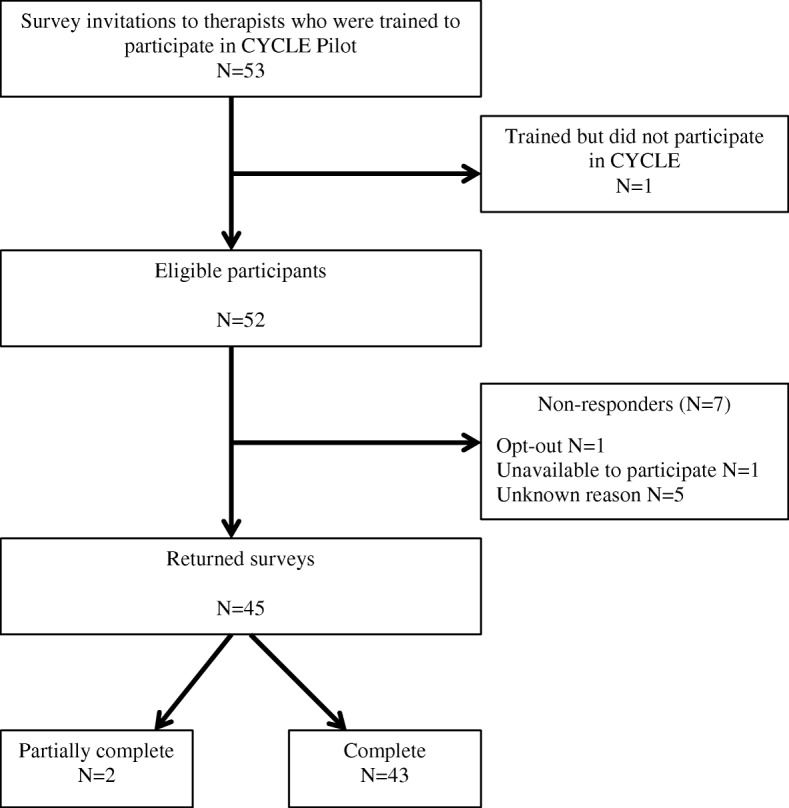
Flow diagram of survey respondents

Of 53 potential participants, 45 completed demographics and at least 1 section, for an 85% response rate (Fig. 1). Sixty-seven percent of respondents practiced in Canada, 87% were PTs, and 91% were female. Most (69%) reported < 10 years of total practice experience and 80% reported < 10 years of experience in the ICU. The median (1st, 3rd quartiles) shift length in the ICU was 7.5 (6, 8) h, and therapists reported a median caseload of 9 (6, 10) patients/shift. Thirty-nine respondents (87%) reported previous research participation; however, only 8 (18%) had formal research training. Twenty respondents (44%) indicated previous experience with in-bed cycling, varying from 0 to 5 (mean [standard deviation (SD)] 1 [1.7]) years. Thirty-nine (87%) respondents indicated previous outcome measure experience, varying from 0 to 25 (mean [SD] 5.7 [5.6]) years. Table 1 summarizes demographic characteristics. Many items demonstrated high scores (i.e., 6 or 7); therefore, we focused on optimizing barriers.

**Table 1:** Demographics of included respondents

Characteristic	Value
Site, *n* respondents/site (% total)	
1	6 (13.3)
2	5 (11.1)
3	5 (11.1)
4	4 (8.9)
5	3 (6.7)
6	2 (4.4)
7	3 (6.7)
8	2 (4.4)
9	5 (11.1)
10	10 (21.2)
Country, *n* sites (% total)	
Canada	8 (80.0)
USA	1 (10.0)
Australia	1 (10.0)
Therapy service delivery model, *n* (%)	
Department-based	22 (48.9)
Program management	18 (40.0)
Matrix model	5 (11.1)
Sex, female, *n* (%)	41 (91.1)
Age, years, *n* (%)	
< 25	0 (0.0)
26–30	13 (28.9)
31–35	13 (28.9)
36–40	11 (24.4)
41–45	3 (6.7)
46–50	5 (11.1)
50+	0 (0.0)
Clinical background, *n* (%)	
Physiotherapist	39 (86.7)
Occupational therapist	5 (11.1)
Physio/occupational therapy assistant	1 (2.2)
Highest level of clinical education, *n* (%)	
Bachelors	16 (35.6)
Masters	26 (57.8)
Doctor of Physical Therapy	3 (6.7)
Country of clinical degree, *n* (%)	
Canada	30 (66.7)
USA	10 (22.2)
Australia	5 (11.1)
Years of therapy practice experience overall, years, *n* (%)	
< 5	17 (37.8)
5–9	14 (31.1)
10–14	6 (13.3)
15–19	3 (6.7)
20+	5 (11.1)
Years of therapy practice experience in ICU, years, *n* (%)	
< 5	21 (46.7)
5–9	15 (33.3)
10–14	5 (11.1)
15–19	3 (6.7)
20+	1 (2.2)
Area of clinical practice, n (%)	
ICU	18 (40.0)
ICU and ward	23 (51.1)
ICU and ward weekends only	3 (6.7)
Other^a^	1 (2.2)
Formal research training, *n* (%)	
Yes	8 (17.8)
Masters	7 (87.5)
Doctorate	1 (12.5)
Previous participation in research, *n* (%)	39 (86.7)
Role in CYCLE^b^, *n* (%)	
Cycling	40 (88.9)
Outcome measures in ICU	30 (66.7)
Blinded outcome measures	11 (24.4)
Study coordination	8 (17.8)

This table describes survey respondent characteristics

*n* number, *ICU* intensive care unit

^a^Primary area of practice was Occupational Therapy team management

^b^Thirty-two (71.1%) respondents reported > 1 role in the study; of the 13 reporting 1 role, 10 conducted cycling and 3 conducted outcome measures (1 ICU outcome measures only, 2 blinded outcome measures only)

### Rehabilitation Practice and Research

**Fig. 2:**
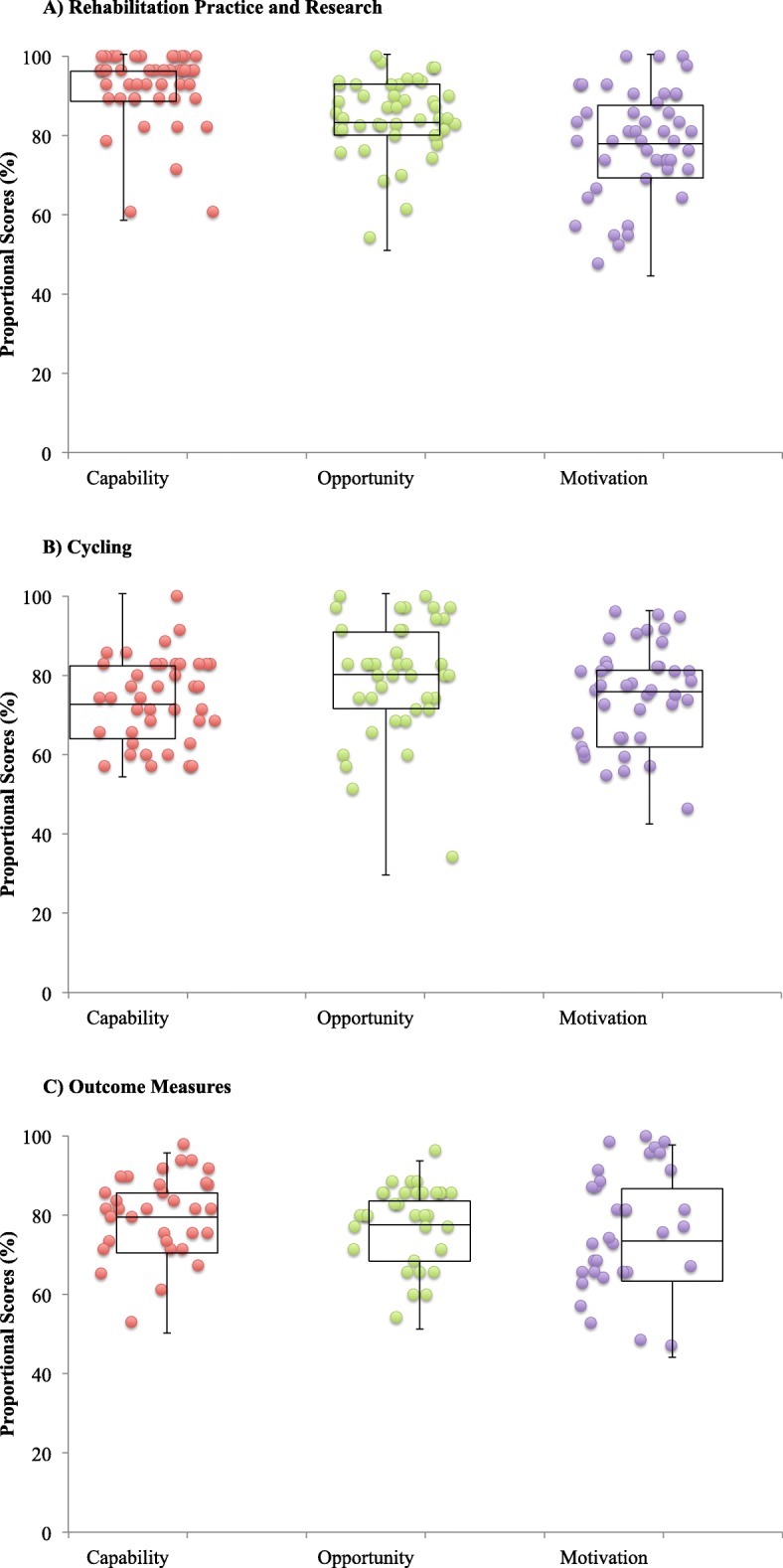
Distribution of respondents’ median proportional scores for items contributing to Capability, Opportunity, and Motivation for **a** Rehabilitation Practice and Research (*n* = 45), **b** Cycling (*n* = 40), and **c** Outcome Measures (*n* = 33). Box plots represent the median score across all respondents’ (horizontal line) and 1st and 3rd quartiles (top and bottom of the box, respectively)

Forty-five (87%) respondents completed this section, which included 20 Likert-type questions (all COM-B: 4 Capability, 10 Opportunity, 6 Motivation). The median overall score was 85% (119/140; [80%, 89%]). The median scores for Capability, Opportunity, and Motivation were as follows: *Capability* 96% (27/28; [89%, 96%]), *Opportunity* 84% (59/70; [81%, 93%]), and *Motivation* 79% (33/42; [71%, 88%]) (Fig. 2a). Additional file 1: Table S3 details individual item scoring.

**Fig. 3:**
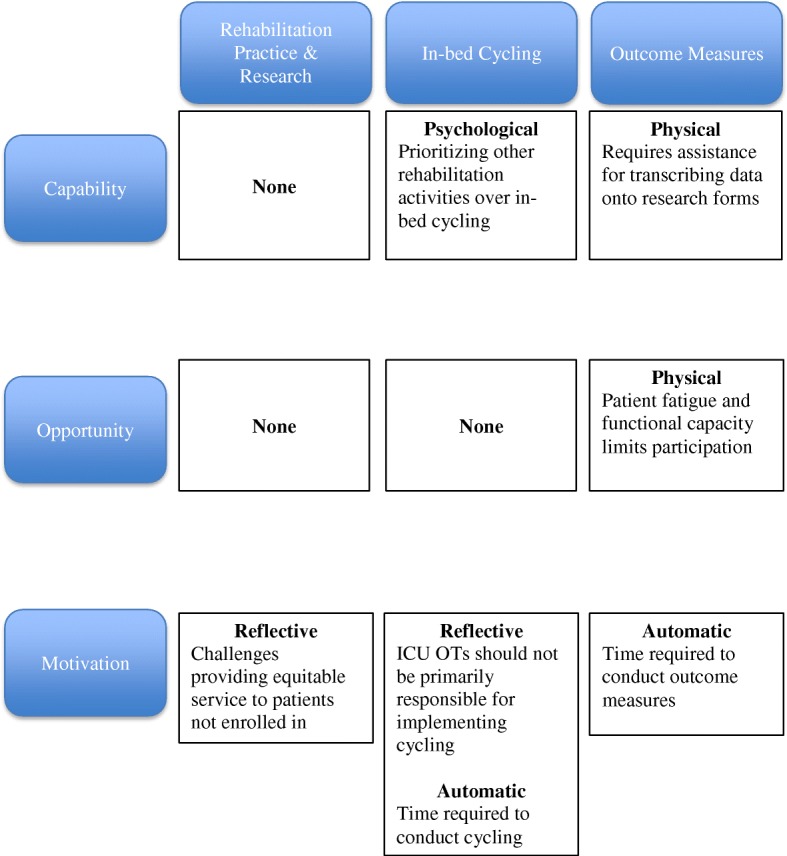
Therapist-reported barriers to participating in conduct of clinical research in the ICU, implementation of early cycling, and conduct of outcome measures

We identified one TDF domain barrier related to Motivation, *beliefs about consequences* (Fig. 3). Therapists reported concerns about providing equitable service to all patients when a patient was enrolled in CYCLE (question 1.3.6, median 3 [2, 3]). The free-text comments supported time as a common theme with concerns regarding time to provide “adequate” treatment for non-study patients and the opportunity cost of cycling (i.e., insufficient time for additional activities therapists felt would be beneficial, e.g., sitting at the edge of the bed).

### Cycling

Forty (89%) respondents reported conducting cycling sessions. This section included 46 questions (22 COM-B, 5 Capability, 5 Opportunity, 12 Motivation). The overall median score for COM-B questions was 77% (119/154; [69%, 83%]). The median scores for Capability, Opportunity, and Motivation were as follows: *Capability* 74% (26/35; [66%, 83%]), *Opportunity* 81% (29/35; [73%, 91%]), and *Motivation* 77% (65/84; [64%, 82%]) (Fig. 2b). Additional file 1: Table S4 details individual item scoring.

We identified barriers in Capability and Motivation. In Capability, the TDF domain *memory*, *attention*, and *decision processes* (Fig. 3) identified therapists’ attitudes towards prioritizing cycling over other activities (question 2.3.5; median 2 [2, 4]). In Motivation, we identified barriers in two TDF domains—*professional role identity* and *emotion*. *Professional role identity* addressed the role of ICU OTs for conducting cycling (question 2.5.2; median 3 [3, 5]; Additional file 1: Table S5). *Emotion* identified concerns with the time required to conduct cycling (question 2.4.3; median 3 [2, 5]).

### Outcome Measures

Thirty-three (73%) respondents reported conducting physical function outcome measures. This section included 24 questions (22 COM-B: 7 Capability, 5 Opportunity, 10 Motivation). The overall median score for COM-B questions was 78% (120/154; [70%, 87%]). The median scores for Capability, Opportunity, and Motivation were as follows: *Capability* 82% (40/49; [73%, 88%]), *Opportunity* 80% (28/35; [71%, 86%]), and *Motivation* 76% (53/70; [66%, 89%]) (Fig. 2c). Additional file 1: Table S6 details individual item scoring.

We identified barriers in Capability, Opportunity, and Motivation (Fig. 3). In Capability, the TDF domain was *skills*, referring to assistance required to transcribe data on research forms (question 3.2.4, median 3 [2, 5]). In Opportunity, the TDF domain was *environmental context and resources*, identifying patients’ fatigue or functional capacity limiting conduct of cycling and outcome measures on the same day (Question 3.3.4, median 2 [2, 3]). In Motivation, the TDF domain was *emotion*, with respondents identifying concerns regarding the time required to conduct outcome measures (Question 3.4.3, median 3 [2, 6]). The free-text responses reinforced these barriers, including patient fatigue requiring multiple attempts to conduct cycling and outcome measures in the same day, time constraints, and challenges transcribing data, particularly when patients were in isolation.

### Comparison of the three sections

**Fig. 4:**
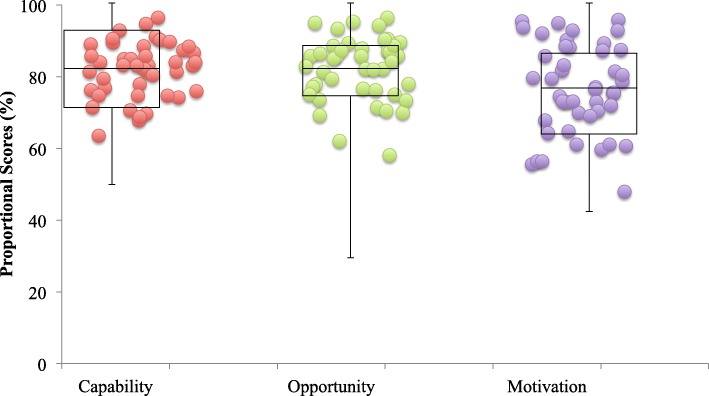
Distribution of respondents’ median proportional scores for items contributing to Capability, Opportunity, and Motivation across the survey. Box plots represent the median score across all respondents (horizontal line) and 1st and 3rd quartiles (top and bottom of the box, respectively)

The Rehabilitation Practice and Research section (85%; 95% confidence interval (CI) [82%, 87%]) was the highest of all three sections (Cycling (77%; 95% CI [73%, 80%]); Outcome Measures (78%; 95% CI [75%, 82%]). Of COM-B attributes, Motivation (77%; 95% CI [73%, 80%]) was lowest, compared to Capability (83%; 95% CI [80%, 84%]) and Opportunity (83%; 95% CI [80%, 85%]) (Fig. 4). Across the three sections, Capability was highest in Rehabilitation Practice and Research versus Cycling (96% vs. 74%) and Outcome Measures (96% vs. 82%). Capability was lower for Cycling versus Outcome Measures (74% vs. 82%). Table 2 summarizes scores by section and by Capability, Opportunity, and Motivation.

**Table 2:** Summary of scores (median proportion (%) and 1st and 3rd quartiles) by survey section and by COM-B attribute

COM-B attribute	Survey section			Overall by COM-B attribute
	Rehabilitation Practice and Research	Cycling	Outcome Measures	
Capability	96.4% (89.3, 96.4)	74.3% (65.7, 82.9)	81.6% (73.5, 87.8)	82.9% (73.5, 92.9)
Opportunity	84.3% (81.4, 92.9)	81.4% (72.9, 91.4)	80.0% (71.4, 85.7)	82.9% (76.2, 88.6)
Motivation	78.6% (71.4, 88.1)	76.8% (64.3, 82.1)	75.7% (65.7, 88.6)	77.7% (65.7, 87.1)
Overall by section	85.0% (80.3, 89.3)	77.3% (68.6, 83.4)	78.0% (70.1, 87.0)	80.9% (74.0, 87.9)

Summary of scores by section and by Capability, Opportunity, and Motivation

## Discussion

We conducted an international, electronic survey of frontline ICU therapists’ perspectives of participation in a multi-center pilot RCT of a novel early rehabilitation technology. We achieved a high response rate and identified keen interest in clinical research participation. Using the theoretically driven COM-B KT framework, Motivation was consistently lowest across the survey. By TDF domain, we identified six barriers for future consideration: Motivation (professional role identity, equitable therapy for non-trial patients, time to conduct cycling or outcome measures), Capability (prioritizing cycling, assistance completing research forms), and Opportunity (patient fatigue). Table 3 outlines barriers by TDF domain and our suggested strategies for optimization in the future RCT.

**Table 3:** Overview of TDF barrier domains (organized by COM-B attribute) and potential strategies

COM-B	Survey section	TDF domain	Barrier	Proposed strategy
Capability	In-bed cycling	Memory/attention/decision processes	I would not cycle with a study patient if I believed other rehabilitation activities were a higher priority for them.	Education re: ethical imperative to deliver randomized intervention. Also related to time barrier (see strategies under “Motivation” attribute below).
	Outcome measures	Skills	I required assistance from another person to transcribe data on research forms during outcome measure assessments.	Training on data form completion. Consider enlisting help from RC to transcribe data during OM assessments, especially for patients in isolation (when unable to bring data forms into room due to infection control limitations).
Opportunity	Outcome measures	Environmental context	Patients’ functional capacity or fatigue limited conduct of cycling and outcome measures on the same day.	Consider conducting OM before cycling, as more active participation required for OM. Consider use of OM as routine care, instead of a separate session.
Motivation	Rehabilitation practice and research	Beliefs about consequences	Implementing the CYCLE protocol presented challenges to providing equitable service for all patients (i.e., patients not enrolled in CYCLE).	Lack of consensus on types of rehabilitation interventions therapists should administer with patients. More local context from therapists is needed to properly address this barrier.
	In-bed cycling	Professional role	If cycling improved outcomes and became recommended standard practice, ICU OTs should not be primarily responsible for implementing this intervention.	Consider OT availability and roles in ICU setting.
	In-bed cycling	Emotion	I felt overwhelmed by the time required to conduct cycling sessions.	Researchers* designing trials include budget for additional therapist time to conduct the research intervention. Therapists support colleagues’ caseloads when a patient is enrolled. Hospital management** incorporating dedicated research time into budgets.
	Outcome measures	Emotion	I felt overwhelmed by the time required to collect outcome measures.	See suggestions under “cycling”. In addition, researchers could consider hybrid model of research whereby therapists conduct cycling and research personnel conduct OM. Therapists could consider using OM as the routine care intervention on days when patients are eligible.

*COM-B* Capability-Opportunity-Motivation-Behaviour system, *TDF* Theoretical Domains Framework, *RPR* rehabilitation practice and research, *OM* outcome measures, *RC* research coordinator, *ICU* intensive care unit, *OT* occupational therapist

*Researchers should consider an integrated approach and collaborate with ICU therapists and individual sites in the design stage. By engaging local expertise at the outset, teams can identify relevant strategies to optimize implementation

**Hospital leaders and management could consider their role in facilitating research; in addition to budget considerations, conveying a positive attitude towards research encourages multidisciplinary coordination and teamwork to conduct the study

Despite the growing interest in ICU rehabilitation interventions, and the critical role of therapists, to our knowledge, the perspectives of frontline therapists involved in research interventions are unknown. We engaged frontline ICU therapists in CYCLE for their specialized clinical expertise conducting rehabilitation interventions with complex, critically ill patients [27] and to optimize the external validity of implementation in different ICUs in preparation for the full RCT. Ideally, pilot work precedes large RCTs to assess feasibility (e.g., recruitment, retention, and implementation) and refine interventions, assessments, or other study procedures [28]. The CYCLE Pilot RCT identified therapist workload as the primary reason for missed cycling sessions on eligible days (*n* = 16 of 38 missed sessions) [18]. To plan for the large RCT, it is imperative to optimize intervention implementation; to do so, we believed it was critical to understand clinicians’ attitudes towards conducting research procedures.

### Rehabilitation Practice and Research

Frontline ICU therapists from our survey were enthusiastic to contribute towards research, which confirms previous work. Several studies using both quantitative [27] and qualitative methodologies [24, 29], in different settings (e.g., ICU [27], acute care [24, 29]), with different patient populations (e.g., ICU [27], early post-stroke [24], multiple sclerosis (MS) [29]), and types of interventions (e.g., a novel post-stroke early mobility [24], balance [29]) documented therapists’ eagerness to be involved with research. Therapists perceived value in the opportunities for professional growth (e.g., developing skills to pursue further education) and personal benefit (e.g., receiving acknowledgments on manuscripts).

While our respondents enjoyed participating in CYCLE, they also expressed concerns about providing equitable service to all patients on their caseload. This finding confirms similar concerns from two other acute care rehabilitation studies [24, 29]. In one study of a balance intervention for patients with MS, PTs reported negative feelings because of the prioritization of time to conduct study-related procedures over care for other patients on their caseload [29]. They also perceived tension between themselves and their supervisors and colleagues [29]. In another study of early mobility for patients post-stroke, PTs felt conflicted about the time required for the research intervention due to their desire to provide equitable service to all patients [24]. Further information about how therapists allocate their time and the types of interventions therapists believe are important for their patients are needed. In addition, future studies involving frontline therapists in research protocols could consider an integrated knowledge translation approach to engage PTs in study design phases and develop active strategies for caseload management with participating sites.

### Cycling

In-bed cycling is a novel intervention gaining popularity in ICU rehabilitation trials [18, 25, 30–38]. However, this advanced technology is not commonly available in ICUs [7] and most of our ICU therapists learned cycling as a new skill. We did not identify knowledge or skills barriers related to cycling.

We identified Capability- and Motivation-related barriers. In Capability, respondents reported they would not prioritize cycling if they perceived another rehabilitation activity was more important. This finding supports a qualitative study where ICU clinicians stated they would not prioritize an intervention if they did not believe there was sufficient supporting evidence [12]. At the time the CYCLE Pilot RCT occurred, two seminal RCTs supported rehabilitation interventions with critically ill patients. One RCT, published in a critical care journal, evaluated in-bed cycling initiated 2 weeks into a patients’ ICU stay and reported better 6-min walk distances for the cycling group at hospital discharge [30]. The second RCT, published in a high-impact general medical journal, evaluated early PT/OT mobility interventions in MV patients and reported improved function at hospital discharge for the intervention group [39]. While our cohort was aware of the evidence for cycling, they may have prioritized the latter evidence. In Motivation, respondents identified concerns about the time required to conduct cycling. Our finding adds to a previous report where 82% of ICU PTs implicated time as the number one barrier to engaging in research [27].

### Outcome Measures

CYCLE used standardized outcome measures to assess differences in physical function between groups. We identified Motivation and Opportunity barriers. Similar to cycling, Motivation was related to time required to conduct outcome measures. Our results support previous research from inpatient, outpatient, and private practice settings, where time was the most commonly reported barrier to conducting outcome measures in the context of high clinical demands [40–43]. Compared to other settings, outcome measures in the ICU may require more time due to patient characteristics including severity of illness, presence of complex catheters and airways, sedation levels, and impaired cognition [44].

The Opportunity barrier identified patient fatigue and/or functional capacity affecting therapists’ ability to conduct both cycling and outcome measures in the same day. In the CYCLE protocol, patients randomized to cycling also required outcome measure assessment at two occasions: ICU awakening and ICU discharge. Therefore, patients received 30 min of cycling and routine PT, which could include strength and physical function outcome measures, in a single day. For activities dependent on patient effort, functional capacity, alertness, motivation, pain levels, or fatigability impact performance [44, 45]. Previous studies highlighted participant fatigue as an important factor, including a RCT that intended to provide 90 min/day of rehabilitation but only achieved 23 min [11]. Importantly, this barrier could impact therapists’ time, requiring > 1 session to conduct cycling and outcome measures to fulfill the study protocol. To minimize this barrier, we chose functional outcome measures with items typically included as part of routine rehabilitation sessions (e.g., strength assessment, transfers, and endurance) [46, 47].

### Implications

We identified Motivation-related barriers in all three survey sections. These included the time required to conduct cycling and outcome measures and perceived challenges providing equitable service to patients who were not enrolled in the study. We believe these are universal barriers for ICU rehabilitation studies and any rehabilitation interventions involving frontline clinicians.

Our respondents reported a median shift length and caseload of 7.5 h and 9 patients per day, respectively. Assuming a 30-min break, this allows approximately 45 min/patient, which includes chart review, communicating and coordinating with the team for rehabilitation (e.g., patient stability, timing of tests, procedures), direct patient care, and documentation. Given this pressing schedule, it is essential that we consider how we can optimize therapists’ time to engage in research-related activities. With the complexity of rehabilitation interventions with critically ill patients, a time-motion study of therapists’ daily workload may identify opportunities to improve efficiencies. For example, common inefficiencies identified in acute care nursing included searching for orders, illegible handwriting, and searching for equipment [48, 49]. Similar studies are urgently needed in the ICU setting with rehabilitation personnel.

Equitable service in the ICU is a complex construct and requires further exploration. Current guidelines from the Society of Critical Care Medicine conditionally recommend that critically ill adults receive rehabilitation/ mobilization interventions in the ICU [50]. However, intervention heterogeneity limited more specific recommendations about the timing, type, or duration of rehabilitation [50]. With the lack of consensus on the types of rehabilitation interventions indicated for ICU patients, we need more enriched information about therapists’ perceptions than available from the survey. For example, more information about patient populations, typical interventions, and the timing and duration of rehabilitation in ICUs is needed.

Engaging frontline therapists represents an important opportunity to optimize implementation of research interventions. Different stakeholders can facilitate engagement. For example, researchers could engage therapists at participating centers in the study design phase or at protocol implementation to identify and minimize barriers. Therapists involved in research could consider how to support each other during the study (e.g., providing caseload support), and hospital leaders and management could consider their roles, including providing dedicated time for staff to take part in research and encouraging interdisciplinary teamwork to implement protocols. In Table 3, we suggest specific ways to facilitate engagement in complex rehabilitation research interventions in ICU.

### Limitations and strengths

Our study has limitations. While quantitative surveys can elicit important factual information, their purpose is to measure specific constructs and is limited in the contextual information they can provide [51]. In follow-up, a qualitative study would be beneficial to gain more in-depth understanding of respondents’ motivations and thinking [51]. As with any survey, there is potential for response bias [52], and we are unable to determine if there were differences between responders and non-responders. For some questions, it would be helpful to understand if respondents had different perspectives based on a patient’s randomized treatment allocation. Patients randomized to cycling received more rehabilitation time than those in routine care in the CYCLE pilot RCT [18]; perceptions of providing equitable service to all patients may have differed by randomized group. This information would help us more effectively address specific barriers. Finally, because our survey was specific to in-bed cycling, it may not be generalizable to all rehabilitation research interventions. However, it provides guidance to others implementing complex interventions, particularly with novel technology.

Our study also has several important strengths. To our knowledge, we are the first to conduct an international survey of frontline ICU therapists involved in a rehabilitation intervention trial with novel technology. Our survey was grounded in established KT theory that provides a robust framework for identifying barriers and potential strategies to overcome them. We conducted rigorous testing throughout survey development to optimize the data and included free-text questions in each section to provide context to quantitative data. Finally, due to our persistent follow-up with non-responders, we had a very high response rate (85%), which optimizes the external validity [22]. Our response rate is similar or better than other surveys of ICU PTs reporting response rates from 22 to 87% [7, 53, 54].

## Conclusions

Given the widespread interest in improving patient outcomes and ICU rehabilitation research, it is imperative to consider the implications of involving frontline providers in clinical research. Our survey sought to understand therapists’ perceptions regarding conduct of clinical research and perceived barriers and facilitators to conducting interventions and outcomes in the CYCLE Pilot RCT. Overall, therapists enjoyed involvement in CYCLE and are enthusiastic to participate in research. However, Motivation, specifically time, was an important barrier. With the CYCLE study advancing to the full RCT, results of this study will help us identify appropriate KT strategies to optimize cycling delivery and outcome measure ascertainment. Furthermore, our results provide important insight to researchers designing and evaluating future complex rehabilitation interventions. Conducting research is vital to improving patient care and outcomes; thus, it should be viewed as a collective responsibility to optimize the engagement of clinicians with the relevant expertise at the right time.

## Supplementary information

**Additional file 1:** CYCLE RCT Survey additional file 1. Electronic Supplement for Therapist Perceptions of ICU Rehab Research. This additional file contains the following: Additional survey methods. **Table S1.** Table of specifications. **Table S2.** Details of survey testing. **Table S3.** Summary statistics for items in the Rehabilitation Practice and Research section. **Table S4.** Summary statistics for items in the Cycling section. **Table S5.** Respondent perceptions regarding primary responsibility for implementing cycling. **Table S6.** Summary statistics for items in the Outcome Measures section. Clinical sensibility testing tool. Reference.

**Additional file 2.** CYCLE RCT Survey additional file 2. Electronic Supplement for Therapist Perceptions of ICU Rehab Research. This additional file contains a copy of the electronic survey.

## Data Availability

Data supporting the findings are available and can be requested from the corresponding author.

## Abbreviations

CI: Confidence interval
COM-B: Capability-Opportunity-Motivation-Behaviour framework
ICU: Intensive care unit
KT: Knowledge translation
MS: Multiple sclerosis
MV: Mechanical ventilation
OT: Occupational therapist
PT: Physiotherapist
RCT: Randomized clinical trial
SD: Standard deviation
TDF: Theoretical Domains Framework
