# Supplementary material for: Therapist perceptions of a rehabilitation research study in the intensive care unit: a trinational survey assessing barriers and facilitators to implementing the CYCLE pilot randomized clinical trial
Source: Pilot Feasibility Stud. 2019 Nov 12;5:131. doi: 10.1186/s40814-019-0509-3 (PMC6849178; doi:10.1186/s40814-019-0509-3)
Supplement: Supplementary file 1 — Additional file 1: CYCLE RCT Survey additional file 1. Electronic Supplement for Therapist Perceptions of ICU Rehab Research. This additional file contains the following: Additional survey methods. Table S1. Table of specifications. Table S2. Details of survey testing. Table S3. Summary statistics for items in the Rehabilitation Practice and Research section. Table S4. Summary statistics for items in the Cycling section. Table S5. Respondent perceptions regarding primary responsibility for implementing cycling. Table S6. Summary statistics for items in the Outcome Measures section. Clinical sensibility testing tool. Reference. [file 40814_2019_509_MOESM1_ESM.docx]

**Title:** Therapist Perceptions of a Rehabilitation Research Study in the Intensive Care Unit: A Trinational Survey Assessing Barriers and Facilitators to Implementing the CYCLE Pilot Randomized Clinical Trial.

**Authors:**

Julie C Reid MSc PT, PhD

Devin McCaskell BSc, MMASc candidate

Michelle E Kho PT, PhD

**Additional File 1**

**Additional File 1**

**Additional Survey Methods:**

**Item generation and reduction –** We developed a table of specifications (**Table E1**) during item generation to ensure each of the 14 TDF domains was represented at least once in the survey. We continued item generation until no new items emerged. We ensured that each section of the survey included items related to Capability, Opportunity, and Motivation. We reduced items with the assistance of the CYCLE Methods Center team by identifying redundant questions and questions perceived to be of limited relevance.

**Question stems** – We developed question stems that addressed a single construct and contained ≤20 words [E1]. Where appropriate, we utilized dynamic questioning to enhance completion by filtering questions according to certain responses (e.g., if “yes” was selected, subsequent “please specify” short answer boxes appeared, etc.). At the end of each survey section, free-text boxes offered respondents opportunities to expand on previous answers or include information that was not already elicited.

**Responses** – Closed-ended questions included either a binary (yes/no) response or ordinal responses using a 7-point Likert-type scale. Most questions were framed positively (e.g., conducting outcome measures is part of my professional role) and assessed respondents’ agreement from strongly disagree (1) to strongly agree (7). We provided “not applicable” response options in case certain questions were not relevant to certain respondents. Participants could review and change their responses using a “previous” button. Participants were also able to save their responses and return to the survey at another time to complete and submit it.

**Survey administration** – All potential respondents received a personalized pre-survey notification letter by email [E1], and 2 business days later, received a personalized email to access the online survey website (<https://surveys.mcmaster.ca/limesurvey>). We sent electronic reminders to non-respondents after 2, 3, and 4 weeks. Since we distributed the survey in late June 2018, corresponding to peak vacation time in North America, we sent a final reminder to non-respondents in early September 2018. Participants could opt-out of the survey via a link in the invitation email.

**Statistical Analysis -** If respondents did not answer up to 2 of the TDF-related Likert scale questions in a section, we imputed a score based on their other responses in that section. If a respondent did not answer >2 questions in a section, we excluded that individual’s section data.

**Table E1.** Table of specifications

| **Questions** | **COM-B Attributes** | | | | | | | | | | | | | |
| --- | --- | --- | --- | --- | --- | --- | --- | --- | --- | --- | --- | --- | --- | --- |
|  | **Capability** | | | | **Opportunity** | | **Motivation** | | | | | | | |
|  | *Physical* | *Psychological* | | | *Social* | *Physical* | *Reflective* | | | | | | *Automatic* | |
|  | **Theoretical Domains Framework** | | | | | | | | | | | | | |
|  | 1. Skills | 1. Knowledge | 1. Memory, attention, decision processes | 1. Behavioural regulation | 1. Social influences | 1. Environmental context and resources | 1. Professional role identity | 1. Beliefs about capabilities | 1. Beliefs about consequences | 1. Intent | 1. Goals | 1. Optimism | 1. Reinforcement | 1. Emotion |
| **Rehabilitation Research and Practice** |  |  |  |  |  |  |  |  |  |  |  |  |  |  |
| 1.1.1) I understand the need for rehabilitation in critically ill patients |  | X |  |  |  |  |  |  |  |  |  |  |  |  |
| 1.1.2) I routinely conduct rehabilitation interventions with critically ill intubated patients |  |  |  | X |  |  |  |  |  |  |  |  |  |  |
| 1.1.3) I routinely conduct rehabilitation interventions with critically ill sedated patients |  |  |  | X |  |  |  |  |  |  |  |  |  |  |
| 1.1.4) Applying research findings to improve practice is part of my professional role |  |  |  |  |  |  | X |  |  |  |  |  |  |  |
| 1.2.1) My site’s organizational culture was supportive of participation in the CYCLE study |  |  |  |  | X |  |  |  |  |  |  |  |  |  |
| 1.2.2) My therapy colleagues in the ICU were supportive of the CYCLE study |  |  |  |  | X |  |  |  |  |  |  |  |  |  |
| 1.2.3) Nurses in the ICU were supportive of the CYCLE study |  |  |  |  | X |  |  |  |  |  |  |  |  |  |
| 1.2.4) Physicians in the ICU were supportive of the CYCLE study |  |  |  |  | X |  |  |  |  |  |  |  |  |  |
| 1.2.5) My manager was supportive of the CYCLE study |  |  |  |  | X |  |  |  |  |  |  |  |  |  |
| 1.2.6) My professional practice leader was supportive of the CYCLE study |  |  |  |  | X |  |  |  |  |  |  |  |  |  |
| 1.3.1) My caseload prevented enrolling patients in CYCLE even when we were fully staffed |  |  |  |  |  | X |  |  |  |  |  |  |  |  |
| 1.3.2) Prior to approaching patients for consent, my opinion was sought regarding their appropriateness for CYCLE |  |  |  |  | X |  |  |  |  |  |  |  |  |  |
| 1.3.3) Prior to approaching patients for consent, my opinion was sought regarding my capacity to offer CYCLE |  |  |  |  | X |  |  |  |  |  |  |  |  |  |
| 1.3.4) I planned my day to facilitate my participation in CYCLE |  |  |  | X |  |  |  |  |  |  |  |  |  |  |
| 1.3.5) When patients were enrolled in CYCLE, I was able to incorporate cycling and outcome measures into my usual caseload |  |  |  |  |  |  |  | X |  |  |  |  |  |  |
| 1.3.6) Implementing the CYCLE Protocol presented challenges to providing equitable service for all patients (i.e., patients not enrolled in CYCLE) |  |  |  |  |  |  |  |  | X |  |  |  |  |  |
| 1.3.7) Having a CYCLE champion (e.g., PT and/or RN or study investigator) in the unit would be important to implementing the CYCLE protocol in our ICU |  |  |  |  | X |  |  |  |  |  |  |  |  |  |
| 1.4.1) When a patient was enrolled in CYCLE, ensuring they received their intervention or outcomes assessment was a high priority |  |  |  |  |  |  |  |  |  |  | X |  |  |  |
| 1.4.2) I enjoyed being part of the CYCLE study |  |  |  |  |  |  |  |  |  |  |  |  |  | X |
| 1.4.3) If the result of the CYCLE study was positive, I would consider cycling as a potential intervention to use with my patients |  |  |  |  |  |  |  |  |  | X |  |  |  |  |
| **In-bed Cycling** |  |  |  |  |  |  |  |  |  |  |  |  |  |  |
| 2.1.1) I am aware of the evidence for cycling with critically ill patients |  | X |  |  |  |  |  |  |  |  |  |  |  |  |
| 2.1.2) I believe that starting cycling early in ICU will improve patient outcomes at, or immediately post, ICU discharge |  |  |  |  |  |  |  |  | X |  |  |  |  |  |
| 2.1.3) I believe that starting cycling early in ICU will improve patient outcomes at hospital discharge |  |  |  |  |  |  |  |  | X |  |  |  |  |  |
| 2.2.1) The bike was easily accessible |  |  |  |  |  | X |  |  |  |  |  |  |  |  |
| 2.2.2) The tablet worked reliably |  |  |  |  |  | X |  |  |  |  |  |  |  |  |
| 2.2.3) I had the technical skills to use the bike | X |  |  |  |  |  |  |  |  |  |  |  |  |  |
| 2.2.4) I felt confident in my ability to cycle with critically ill patients |  |  |  |  |  |  |  | X |  |  |  |  |  |  |
| 2.2.5) I could collect data (e.g., vital signs, cycling information) during a therapy session | X |  |  |  |  |  |  |  |  |  |  |  |  |  |
| 2.2.6) I required assistance from another person to transcribe data on research forms during a therapy session | X |  |  |  |  |  |  |  |  |  |  |  |  |  |
| 2.3.1) If we were fully staffed, we could consistently deliver cycling sessions to patients enrolled in CYCLE |  |  |  |  |  | X |  |  |  |  |  |  |  |  |
| 2.3.2) Our team’s communication facilitated consistent delivery of cycling sessions with enrolled patients |  |  |  |  | X |  |  |  |  |  |  |  |  |  |
| 2.3.3) Our team’s coordination facilitated consistent delivery of cycling sessions with enrolled patients |  |  |  |  | X |  |  |  |  |  |  |  |  |  |
| 2.3.4) When patients were enrolled in CYCLE, I felt an ethical responsibility to deliver the cycling intervention on all eligible days |  |  |  |  |  |  | X |  |  |  |  |  |  |  |
| 2.3.5) I would not cycle with a patient if I believed other rehabilitation activities were a higher priority for them |  |  | X |  |  |  |  |  |  |  |  |  |  |  |
| 2.4.1) I felt overwhelmed by cycling with critically ill patients |  |  |  |  |  |  |  |  |  |  |  |  |  | X |
| 2.4.2) I felt overwhelmed by the bike set-up and takedown |  |  |  |  |  |  |  |  |  |  |  |  |  | X |
| 2.4.3) I felt overwhelmed by the time required to conduct cycling sessions |  |  |  |  |  |  |  |  |  |  |  |  |  | X |
| 2.4.4) I had anxiety related to cycling with critically ill patients |  |  |  |  |  |  |  |  |  |  |  |  |  | X |
| 2.4.5) If I had a safety event during a cycling session, I would be reluctant to cycle again in the future |  |  |  |  |  |  |  |  |  |  |  |  | X |  |
| 2.5.1) ICU PTs should be responsible for implementing cycling |  |  |  |  |  |  | X |  |  |  |  |  |  |  |
| 2.5.2) ICU OTs should be responsible for implementing cycling |  |  |  |  |  |  | X |  |  |  |  |  |  |  |
| 2.7) How important is it for you to lead cycling sessions with patients enrolled in CYCLE? |  |  |  |  |  |  | X |  |  |  |  |  |  |  |
| **Outcome Measures** |  |  |  |  |  |  |  |  |  |  |  |  |  |  |
| 3.1.1) Conducting outcome measures with patients is part of my professional role |  |  |  |  |  |  | X |  |  |  |  |  |  |  |
| 3.1.2) Outcome measures reflect important differences in patients’ physical function |  |  |  |  |  |  |  |  | X |  |  |  |  |  |
| 3.1.3) I routinely conduct outcome measures with critically ill patients |  |  |  | X |  |  |  |  |  |  |  |  |  |  |
| 3.1.4) I understand why we used the outcome measures in CYCLE |  | X |  |  |  |  |  |  |  |  |  |  |  |  |
| 3.1.5) The outcome measures used in CYCLE can be incorporated as part of routine therapy in the ICU |  |  |  |  |  |  |  |  |  |  |  | X |  |  |
| 3.2.1) I had the technical skills to conduct the outcome measures used in CYCLE | X |  |  |  |  |  |  |  |  |  |  |  |  |  |
| 3.2.2) I felt confident in my ability to collect the outcome measures used in CYCLE |  |  |  |  |  |  |  | X |  |  |  |  |  |  |
| 3.2.3) I could collect data (e.g., count steps) during outcome measure assessments | X |  |  |  |  |  |  |  |  |  |  |  |  |  |
| 3.2.4) I required assistance from another person to transcribe data on research forms during outcome measure assessments | X |  |  |  |  |  |  |  |  |  |  |  |  |  |
| 3.2.5) The equipment needed for the CYCLE outcome measures was easily accessible |  |  |  |  |  | X |  |  |  |  |  |  |  |  |
| 3.3.1) If we were fully staffed, we could consistently collect outcome measures for patients enrolled in CYCLE |  |  |  |  |  | X |  |  |  |  |  |  |  |  |
| 3.3.2) It was feasible to conduct outcomes assessments and cycling sessions with patients on the same day |  |  |  | X |  |  |  |  |  |  |  |  |  |  |
| 3.3.4) Patients’ functional capacity or fatigue limited conduct of cycling and outcome measures on the same day |  |  |  |  |  | X |  |  |  |  |  |  |  |  |
| 3.3.5) If I had limited time or a patient was tired, I knew which outcome measures to prioritize collecting |  | X |  |  |  |  |  |  |  |  |  |  |  |  |
| 3.3.6) Our team’s communication facilitated consistent collection of CYCLE outcome measures with eligible patients |  |  |  |  | X |  |  |  |  |  |  |  |  |  |
| 3.3.7) Our team’s coordination facilitated consistent collection of CYLCE outcome measures with eligible patients |  |  |  |  | X |  |  |  |  |  |  |  |  |  |
| 3.4.1) I felt overwhelmed by collecting outcome measures with critically ill patients |  |  |  |  |  |  |  |  |  |  |  |  |  | X |
| 3.4.2) I felt overwhelmed by the equipment set-up and takedown needed for the outcome measures |  |  |  |  |  |  |  |  |  |  |  |  |  | X |
| 3.4.3) I felt overwhelmed by the time required to collect outcome measures |  |  |  |  |  |  |  |  |  |  |  |  |  | X |
| 3.4.4) I felt overwhelmed by the number of outcome measures to collect |  |  |  |  |  |  |  |  |  |  |  |  |  | X |
| 3.4.5) I felt anxious when I knew I had to conduct outcome measures with a CYCLE patient |  |  |  |  |  |  |  |  |  |  |  |  |  | X |
| 3.6) I intend to use the outcome measures from CYCLE as part of usual care |  |  |  |  |  |  |  |  |  | X |  |  |  |  |

**Legend:** The survey was divided into 3 main sections: Rehabilitation Practice and Research, In-bed Cycling, and Outcome Measures. Sections were further sub-divided for improved flow. For example, Section 1 was sub-divided into the following: Rehabilitation knowledge and practice, Organizational support for CYCLE, Implementing CYCLE, and Your role in CYCLE. Question numbering followed section and subsections, e.g., questions 1.1.1 to 1.1.4 pertain to rehabilitation knowledge and practice in the Rehabilitation Practice and Research section.

**Table E2.** Details of survey testing stages

| **Testing stage** | **Description** | **Assessors** |
| --- | --- | --- |
| Pre-testing [E1] | Assessed how respondents comprehended survey questions and evaluated the extent questions were interpreted in the same way. Based on feedback, we revised question and answer stems for clarity | -2 experienced acute care PTs  -1 PT student  -1 rehabilitation science MSc student |
| Pilot testing [E1] | Assessed the dynamics of the survey with respect to flow, acceptability, administrative ease. Identified redundant, irrelevant, or poorly worded questions or answer options. We continued testing until no new feedback emerged. Based on feedback, we revised the survey to improve flow and clarity | -5 acute care PTs  -1 research coordinator |
| Clinical sensibility testing* [E1] | Assessed the comprehensiveness, clarity, and face validity of the survey using a structured tool (Online Supplement 3). We continued testing until no new feedback emerged. | -4 research coordinators  -1 research assistant  -1 site principal investigator  -1 PT |

**Legend:** PT – Physiotherapist; * We engaged representatives from each participating country to ensure questions, answer options, and terminology were easily understood and relevant to their practice

**Table E3.** Summary statistics for items in the Rehabilitation Practice and Research section.

| **TDF Domain** | **Item** | **Median (1^st^, 3^rd^)** |
| --- | --- | --- |
| **Overall section score (/140)** | **20 items** | **119 (113, 125) (85%)** |
| **CAPABILITY (/28)** | **4 items** | **27 (25, 27) (96%)** |
| Behavioural Regulation | 3 items | 7 (6, 7) |
|  | 1.1.2 – Rehab on intubated patients | 7 (7, 7) |
|  | 1.1.3 – Rehab on sedated patients | 6 (5, 7) |
|  | 1.3.4 – Plan day to facilitate CYCLE | 7 (6, 7) |
| Knowledge | 1.1.1 – Rehab needs of ICU patients | 7 (7, 7) |
| **OPPORTUNITY (/70)** | **10 items** | **59 (57, 65) (84%)** |
| Social Influences | 9 items | 7 (6, 7) |
|  | 1.2.1 – Organization supports CYCLE | 7 (6, 7) |
|  | 1.2.2 – Therapy staff supports CYCLE | 7 (7, 7) |
|  | 1.2.3 – Nurses support CYCLE | 6 (6, 7) |
|  | 1.2.4 – Physicians support CYCLE | 7 (6, 7) |
|  | 1.2.5 – Manager supports CYCLE | 7 (6, 7) |
|  | 1.2.6 – Professional leader supports CYCLE | 6 (6, 7) |
|  | 1.3.2 – Coordinating re: patient appropriateness | 6 (5, 7) |
|  | 1.3.3 – Coordinating re: staff capacity | 6 (4, 7) |
|  | 1.3.7 – CYCLE champion | 6 (6, 7) |
| Environmental Context | 1.3.1 – Caseload prevents enrolling patients | 5 (3, 6) |
| **MOTIVATION (/42)** | **6 items** | **33 (30, 37) (79%)** |
| Professional Role | 1.1.4 – Evidence-based practice | 7 (6, 7) |
| Emotion | 1.4.2 – Enjoy participating in CYCLE | 6 (5, 7) |
| Beliefs about Consequences | 1.3.6 – Equitable service | 3 (2, 3) |
| Beliefs about Capabilities | 1.3.5 – Incorporating CYCLE | 5 (3, 6) |
| Goals | 1.4.1 – Ensuring CYCLE protocol delivery | 7 (7, 7) |
| Intent | 1.4.3 – Cycling as a routine intervention | 7 (5, 7) |

**Table E4**. Summary statistics for items in the Cycling section.

| **TDF Domain** | **Item** | **Median (1^st^, 3^rd^)** |
| --- | --- | --- |
| **Overall section score (/154)** | **22 items** | **119 (105, 129) (77%)** |
| **CAPABILITY (/35)** | **5 items** | **26 (23, 29) (74%)** |
| Skills | 3 items | 6 (5, 7) |
|  | 2.2.3 – Technical skills | 6 (6, 7) |
|  | 2.2.5 – Data collection | 7 (6, 7) |
|  | 2.2.6 – Assistance for data | 4 (3, 6) |
| Knowledge | 2.1.1 – Aware of evidence | 7 (6, 7) |
| Memory, attention, decision processes | 2.3.5 – Cycling priority | 2 (2, 4) |
| **OPPORTUNITY (/35)** | **5 items** | **29 (26, 32) (81%)** |
| Environmental context | 3 items | 6 (4, 7) |
|  | 2.2.1 – Bike accessibility | 6 (4, 7) |
|  | 2.2.2 – Tablet worked | 5 (3, 6) |
|  | 2.3.1 – Cycling delivery | 6 (5, 7) |
| Social influences | 2 items | 7 (6, 7) |
|  | 2.3.2 – Team communication | 7 (6, 7) |
|  | 2.3.3 – Team coordination | 7 (6, 7) |
| **MOTIVATION (/84)** | **12 items** | **65 (54, 69) (77%)** |
| Professional role | 4 items | 6 (4, 7) |
|  | 2.3.4 – Ethical responsibility | 7 (6, 7) |
|  | 2.5.1 – Cycling responsibility (PT) | 6 (5, 7) |
|  | 2.5.2 – Cycling responsibility (OT) | 3 (3, 5) |
|  | 2.7 – Leading cycling | 5 (4, 6) |
| Emotion | 4 items | 6 (3, 7) |
|  | 2.4.1 – Overwhelmed | 6 (4, 7) |
|  | 2.4.2 – Bike setup/takedown | 5 (3, 6) |
|  | 2.4.3 – Time requirement | 3 (2, 5) |
|  | 2.4.4 – Cycling anxiety | 7 (5, 7) |
| Beliefs about consequences | 2 items | 6 (5, 7) |
|  | 2.1.2 – Cycling improves outcomes | 6 (5, 7) |
|  | 2.1.3 – Outcomes at discharge | 6 (5, 7) |
| Beliefs about capabilities | 2.2.4 – cycling confidence | 6 (6, 7) |
| Reinforcement | 2.4.5 – safety events | 6 (5, 7) |

**Table E5.** Respondent perceptions regarding primary responsibility for implementing cycling

|  | **ICU PT (n, % agreement)** | **ICU OT (n, % agreement)** |
| --- | --- | --- |
| **PT respondents (n=36)** | 27 (75%) | 6 (17%)* |
| **OT respondents (n=4)** | 4 (100%) | 1 (25%) |

**Legend:** * 5 (14%) PT respondents indicated that ICU OTs implementing cycling sessions was not applicable.

**Table E6.** Summary statistics for items represented in the Outcome Measures section.

| **TDF Domain** | **Item** | **Median (1^st^, 3^rd^)** |
| --- | --- | --- |
| **Overall section score (/154)** | **22 items** | **120 (108, 134) (78%)** |
| **CAPABILITY (/49)** | **7 items** | **40 (36, 43) (82%)** |
| Skills | 3 items | 6 (5, 7) |
|  | 3.2.1 – Technical skills | 7 (7, 7) |
|  | 3.2.3 – Data collection | 7 (6, 7) |
|  | 3.2.4 – Assistance for data | 3 (2, 5) |
| Knowledge | 2 items | 7 (6, 7) |
|  | 3.1.4 – Understand CYCLE outcomes | 7 (7, 7) |
|  | 3.3.5 – Prioritizing outcome measures | 6 (6, 7) |
| Behavioural Regulation | 2 items | 5 (3, 6) |
|  | 3.1.3 – Routine use of OM | 6 (5, 6) |
|  | 3.3.2 – Feasible to conduct OM and cycling | 5 (3, 6) |
| **OPPORTUNITY (/35)** | **5 items** | **28 (25, 30) (80%)** |
| Environmental Context | 3 items | 6 (3, 7) |
|  | 3.2.5 – Equipment accessible | 7 (6, 7) |
|  | 3.3.1 – Consistent collection | 7 (6, 7) |
|  | 3.3.4 – Patient capacity limiting factor | 2 (2, 3) |
| Social Influences | 2 items | 7 (6, 7) |
|  | 3.3.6 – Team communication | 7 (6, 7) |
|  | 3.3.7 – Team coordination | 6 (6, 7) |
| **MOTIVATION (/70)** | **10 items** | **53 (46, 62) (76%)** |
| Emotion | 5 items | 5 (3, 6) |
|  | 3.4.1 – Overwhelmed | 6 (4, 7) |
|  | 3.4.2 – Equipment setup/takedown | 6 (3, 7) |
|  | 3.4.3 – Time requirement | 3 (2, 6) |
|  | 3.4.4 – Number of OMs | 4 (3, 6) |
|  | 3.4.5 - Anxious | 5 (4, 7) |
| Professional Role | 3.1.1 – Conducting OM part of my role | 7 (6, 7) |
| Beliefs about Consequences | 3.1.2 – OMs reflect differences | 7 (6, 7) |
| Optimism | 3.1.5 – OM can be part of routine care | 6 (5, 7) |
| Beliefs about Capabilities | 3.2.2 – Confidence with OM | 7 (6, 7) |
| Intent | 3.5 – Intend to use OM | 5 (4, 6) |

**Clinical sensibility testing tool**

We are interested in your opinion of the clinical sensibility of the CYCLE Pilot RCT survey. To help us improve the survey, please answer the following questions:

1. **Cycling (please circle your responses)**

|  | Not at all | | | Completely | | | | |
| --- | --- | --- | --- | --- | --- | --- | --- | --- |
| 1. To what extent do the questions target important issues pertaining to in-bed cycling with critically ill patients? | 1 | 2 | 3 | | 4 | 5 | 6 | 7 |
| 1. To what extent do the questions elicit information pertaining to your experiences cycling with critically ill patients? | 1 | 2 | 3 | | 4 | 5 | 6 | 7 |
|  |  |  |  | |  |  |  |  |
|  | **Crucial issues missing** | | | |  | **No issues missing** | | |
| 1. Are there important issues pertaining to cycling with critically ill patients that are missing from the survey that should be included? | 1 | 2 | 3 | | 4 | 5 | 6 | 7 |

Please identify any omissions: ­­­­­­­­­­­­­­­___________________________________________________________________

1. **Outcome measures (please circle your responses)**

|  | Not at all | | | Completely | | | | |
| --- | --- | --- | --- | --- | --- | --- | --- | --- |
| 1. To what extent do the questions target important issues pertaining to conducting CYCLE outcome measures with critically ill patients? | 1 | 2 | 3 | | 4 | 5 | 6 | 7 |
| 1. To what extent do the questions elicit information pertaining to your experiences with conducting CYCLE outcome measures with critically ill patients? | 1 | 2 | 3 | | 4 | 5 | 6 | 7 |
|  |  |  |  | |  |  |  |  |
|  | **Crucial issues missing** | | | |  | **No issues missing** | | |
| 1. Are there important issues pertaining to conducting CYCLE outcome measures with critically ill patients that are missing from the survey that should be included? | 1 | 2 | 3 | | 4 | 5 | 6 | 7 |

Please identify any omissions: ­­­­­­­­­­­­­­­___________________________________________________________________

1. **Overall (please circle your responses)**

|  | Not at all | | | Completely | | | | |
| --- | --- | --- | --- | --- | --- | --- | --- | --- |
| 1. To what extent are the response options provided simple and easy to understand? | 1 | 2 | 3 | | 4 | 5 | 6 | 7 |
|  |  |  |  | |  |  |  |  |
|  | **Not at all likely** | | | |  | **Extremely likely** | | |
| 1. How likely is the survey to elicit barriers and facilitators to implementing CYCLE with critically ill patients? | 1 | 2 | 3 | | 4 | 5 | 6 | 7 |

|  | Almost all | | |  | None | | |
| --- | --- | --- | --- | --- | --- | --- | --- |
| 1. How many items are inappropriate or redundant? | 1 | 2 | 3 | 4 | 5 | 6 | 7 |

Please identify any redundant or inappropriate items: ­­­­­­­­­­­­_______________________________________________

1. **How long did it take to complete the CYCLE Pilot RCT survey?** ________minutes

Please feel free to provide any additional feedback on this form.

**Reference:**

E1. Burns KE, Duffett M, Kho ME, et al. A guide for the design and conduct of self-administered surveys of clinicians. CMAJ 2008;**179**(3):245-52 doi: 10.1503/cmaj.080372[published Online First: Epub Date]|.
