# Supplementary material for: Therapist perceptions of a rehabilitation research study in the intensive care unit: a trinational survey assessing barriers and facilitators to implementing the CYCLE pilot randomized clinical trial
Source: Pilot Feasibility Stud. 2019 Nov 12;5:131. doi: 10.1186/s40814-019-0509-3 (PMC6849178; doi:10.1186/s40814-019-0509-3)
Supplement: Supplementary file 2 — Additional file 2. CYCLE RCT Survey additional file 2. Electronic Supplement for Therapist Perceptions of ICU Rehab Research. This additional file contains a copy of the electronic survey. [file 40814_2019_509_MOESM2_ESM.pdf]

Title: Therapist Perceptions of Implementing a Novel Rehabilitation Research Study in the Intensive Care Unit: A Trinational survey.

Authors:
Julie C Reid MSc PT, PhD candidate
Devin McCaskell BSc, MMASc candidate
Michelle E Kho PT, PhD

Additional File 2

CYCLE Pilot RCT Survey

The CYCLE Pilot RCT was a study to assess the feasibility of early in-bed cycling with mechanically ventilated patients in multiple ICUs.

We are interested in your experiences with and perceptions of barriers and facilitators to participating in the CYCLE study. Information from this survey will help therapists and research coordinators conduct the future large CYCLE study.

Thank you for your participation.

There are 59 questions in this survey.

Preamble Statement

This survey is administered by Julie Reid (PhD candidate) and Dr. Michelle Kho on behalf of the CYCLE study team. The purpose is to understand your experiences with and perceptions of barriers and facilitators to conducting in-bed cycling and outcome measures as part of CYCLE. Your input is invaluable and what we learn from this survey will help us plan for the larger randomized clinical trial to optimize cycling delivery and outcome measure ascertainment.

This survey has 5 pages and should take approximately 20-25 minutes to complete.

Please refer to the accompanying letter of information (/limesurvey/upload/surveys/838514/files/Reid-letter%20of%20information-FINALmk.pdf) to learn more about the survey, particularly in terms of any associated risks or harms, how confidentiality and anonymity will be handled, withdrawal procedures, and how to obtain information about the survey's results.

This survey is part of a study that has been reviewed and cleared by the Hamilton Integrated Research Ethics Board (HiREB) (http://www.hireb.ca/). The HiREB protocol number associated with this survey is 14-531. You are free to complete this survey or not. If you have any concerns or questions about your rights as a participant or about the way the study is being conducted, please contact HIREB. The HIREB is responsible for ensuring that participants are informed of the risks associated with the research, and that participants are free to decide if participation is right for them. If you have any questions about your rights as a research participant, please call the Office of the Chair, Hamilton Integrated Research Ethics Board at 905.521.2100 ext 42013.

Consent to Participate

Having read the above, I understand that by clicking the “Yes” button below, I agree to take part in this study under the terms and conditions outlined in the accompanied letter of information. *

❶ Choose one of the following answers
Please choose **only one** of the following:

- ◯ Yes, I agree to participate
- ◯ No, I do not agree to participate

What center were you practicing at during your involvement with CYCLE?

Choose one of the following answers
Please choose **only one** of the following:

- St. Joseph's Healthcare Hamilton
- Juravinski Hospital
- Hamilton General Hospital
- Toronto General Hospital
- St. Michael's Hospital
- London Health Sciences
- Ottawa General Hospital
- Ottawa Civic Hospital
- Austin Health
- Duke

Sex

Choose one of the following answers
Please choose **only one** of the following:

- Female
- Male
- Prefer not to say

Age

Choose one of the following answers
Please choose **only one** of the following:

- < 25
- 26 to 30
- 31 to 35
- 36 to 40
- 41 to 45
- 46 to 50
- 51+

What is your clinical background?

❗ Check all that apply

Please choose **all** that apply:

- [ ] Physiotherapist/physical therapist (PT)
- [ ] Occupational therapist (OT)
- [ ] PT/OT assistant
- [ ] Respiratory therapist
- [ ] Registered nurse
- [ ] Physician
- [ ] I have no clinical background
- [ ] Other: ____________________

What is your highest level of clinical education?

❗ Choose one of the following answers

Please choose **only one** of the following:

- ◯ Diploma
- ◯ Bachelors
- ◯ Masters
- ◯ Doctor of Physical (DPT)
- ◯ Doctor of Occupational Therapy (DOT)
- ◯ Other degree(s) (please specify)

Make a comment on your choice here:

Do you have formal research education (e.g., research-based Masters, PhD)?

Please choose **only one** of the following:

- ◯ Yes
- ◯ No

What is your highest level of completed research education?

Only answer this question if the following conditions are met:
Answer was 'Yes' at question '8 [D17]' (Do you have formal research education (e.g., research-based Masters, PhD)?)

❗ Choose one of the following answers
Please choose **only one** of the following:

- ◯ Masters
- ◯ Doctorate

Prior to your involvement in CYCLE, had you previously participated in research?

Please choose **only one** of the following:

- ◯ Yes
- ◯ No

What year did you begin your involvement with CYCLE? (yyyy)

❗ Only numbers may be entered in this field.
Please write your answer here:

What was your role in CYCLE?

❗ Check all that apply
Please choose **all** that apply:

- ☐ Cycling with patients in ICU
- ☐ Physical function assessments in ICU
- ☐ Blinded physical function assessments
- ☐ Study coordination (e.g., recruitment, enrolment, patient-reported outcome measures)

In what country did you receive your clinical degree?

Only answer this question if the following conditions are met:
Answer was 'Cycling with patients in ICU' *or* 'Physical function assessments in ICU' *or* 'Blinded physical function assessments' at question '12 [D03]' (What was your role in CYCLE?)

Choose one of the following answers
Please choose **only one** of the following:

- ◯ Canada
- ◯ Australia
- ◯ United States
- ◯ Other (please specify below)

Make a comment on your choice here:

At the time you started CYCLE, how long had you practiced PT or OT?

Only answer this question if the following conditions are met:
Answer was 'Cycling with patients in ICU' *or* 'Blinded physical function assessments' *or* 'Physical function assessments in ICU' at question '12 [D03]' (What was your role in CYCLE?)

Choose one of the following answers
Please choose **only one** of the following:

- ◯ Less than 5 years
- ◯ 5 - 9 years
- ◯ 10 - 14 years
- ◯ 15 - 19 years
- ◯ 20 + years

At the time you started CYCLE, how long had you practiced PT or OT in the ICU?

Only answer this question if the following conditions are met:
Answer was 'Blinded physical function assessments' *or* 'Cycling with patients in ICU' *or* 'Physical function assessments in ICU' at question '12 [D03]' (What was your role in CYCLE?)

❶ Choose one of the following answers
Please choose **only one** of the following:

- ◯ Less than 5 years
- ◯ 5 - 9 years
- ◯ 10 - 14 years
- ◯ 15 - 19 years
- ◯ 20 + years

At the time of CYCLE, what service delivery model did you work in?

Only answer this question if the following conditions are met:
Answer was 'Physical function assessments in ICU' *or* 'Blinded physical function assessments' *or* 'Cycling with patients in ICU' at question '12 [D03]' (What was your role in CYCLE?)

❶ Choose one of the following answers
Please choose **only one** of the following:

- ◯ Department-based (therapists work for a centralized therapy department and not a specific program)
- ◯ Program management (therapists work for individual programs (e.g., ICU, orthopaedics, general surgery, etc.) and are managed in interdisciplinary teams)
- ◯ Matrix model (therapists work within a specific program and report to the program's manager, but support and leadership are provided by a discipline-specific person)

At the time of CYCLE, what area(s) of the hospital did you practice in?

Only answer this question if the following conditions are met:
Answer was 'Blinded physical function assessments' *or* 'Cycling with patients in ICU' *or* 'Physical function assessments in ICU' at question '12 [D03]' (What was your role in CYCLE?)

❗ Choose one of the following answers
Please choose **only one** of the following:

- ◯ ICU
- ◯ ICU and ward
- ◯ ICU and ward on weekends only
- ◯ ICU on weekends only
- ◯ Wards on weekends only
- ◯ Other (please specify below)

Make a comment on your choice here:

During CYCLE, how long was your typical clinical shift (hours)?

Only answer this question if the following conditions are met:
Answer was 'Blinded physical function assessments' *or* 'Cycling with patients in ICU' *or* 'Physical function assessments in ICU' at question '12 [D03]' (What was your role in CYCLE?)

How many patients did you typically see in your clinical shift?

Only answer this question if the following conditions are met:
Answer was 'Cycling with patients in ICU' *or* 'Blinded physical function assessments' *or* 'Physical function assessments in ICU' at question '12 [D03]' (What was your role in CYCLE?)

Considering your total practice hours in the last year, please indicate the percentage of hours spent in each of the following: (total percentage must not exceed 100)

Only answer this question if the following conditions are met:
Answer was 'Blinded physical function assessments' *or* 'Physical function assessments in ICU' *or* 'Cycling with patients in ICU' at question '12 [D03]' (What was your role in CYCLE?)

❗ Only numbers may be entered in these fields.
Please write your answer(s) here:

Direct patient care services	
Administration (including meetings, continuing education, etc.)	
Research	
Academic teaching to prepare students for a health profession	
Clinical education while providing professional services	
Service to the profession (e.g., service to professional associations outside of hospital, etc.)	
All other areas	

At the time you started CYCLE, how much experience did you have with cycling?

Only answer this question if the following conditions are met:
Answer was 'Blinded physical function assessments' *or* 'Physical function assessments in ICU' *or* 'Cycling with patients in ICU' at question '12 [D03]' (What was your role in CYCLE?)

❗ Only numbers may be entered in these fields.
Please write your answer(s) here:

Years	

Please answer with decimals for partial years (e.g., 6 months = 0.5 years)

At the time you started CYCLE, how much experience did you have conducting outcome measures?

Only answer this question if the following conditions are met:
Answer was 'Physical function assessments in ICU' *or* 'Cycling with patients in ICU' *or* 'Blinded physical function assessments' at question '12 [D03]' (What was your role in CYCLE?)

❗ Only numbers may be entered in these fields.
Please write your answer(s) here:

Years	

Please answer with decimals for partial years (e.g., 6 months = 0.5 years)

1.1. Rehabilitation knowledge and practice

Please choose the appropriate response for each item:

	Strongly disagree	Moderately disagree	Somewhat disagree	Neutral (neither disagree nor agree)	Somewhat agree	Moderately agree	Strongly agree	Not applicable
**1.1.1) I understand the need for rehabilitation in critically ill patients.**	◯	◯	◯	◯	◯	◯	◯	◯
**1.1.2) I routinely conduct rehabilitation interventions with critically ill <u>intubated</u> patients.**	◯	◯	◯	◯	◯	◯	◯	◯
**1.1.3) I routinely conduct rehabilitation interventions with critically ill <u>sedated</u> patients.**	◯	◯	◯	◯	◯	◯	◯	◯
**1.1.4) Applying research findings to improve practice is part of my professional role.**	◯	◯	◯	◯	◯	◯	◯	◯

1.2. Organizational support for CYCLE

Please choose the appropriate response for each item:

	Strongly disagree	Moderately disagree	Somewhat disagree	Neutral (neither agree nor disagree)	Somewhat agree	Moderately agree	Strongly agree	Not applicable
**1.2.1) My site's organizational culture was supportive of participation in the CYCLE study.**	◯	◯	◯	◯	◯	◯	◯	◯
**1.2.2) My therapy colleagues in the ICU were supportive of the CYCLE study.**	◯	◯	◯	◯	◯	◯	◯	◯
**1.2.3) Nurses in the ICU were supportive of the CYCLE study.**	◯	◯	◯	◯	◯	◯	◯	◯
**1.2.4) Physicians in the ICU were supportive of the CYCLE study.**	◯	◯	◯	◯	◯	◯	◯	◯
**1.2.5) My manager was supportive of the CYCLE study.**	◯	◯	◯	◯	◯	◯	◯	◯
**1.2.6) My professional practice leader (may also be known as supervisor) was supportive of the CYCLE study.**	◯	◯	◯	◯	◯	◯	◯	◯

1.3. Implementing CYCLE

Please choose the appropriate response for each item:

	Strongly disagree	Moderately disagree	Somewhat disagree	Neutral (neither agree nor disagree)	Somewhat agree	Moderately agree	Strongly agree	Not applicable
**1.3.1) My caseload prevented enrolling patients in CYCLE even when we were fully staffed.**	◯	◯	◯	◯	◯	◯	◯	◯
**1.3.2) Prior to approaching patients for consent, my opinion was sought regarding their appropriateness for CYCLE.**	◯	◯	◯	◯	◯	◯	◯	◯
**1.3.3) Prior to approaching patients for consent, my opinion was sought regarding my capacity to offer CYCLE (i.e., based on staff availability and caseload).**	◯	◯	◯	◯	◯	◯	◯	◯
**1.3.4) I planned my day to facilitate my participation in CYCLE.**	◯	◯	◯	◯	◯	◯	◯	◯
**1.3.5) When patients were enrolled in CYCLE, I was able to incorporate cycling and outcome measures into my usual caseload.**	◯	◯	◯	◯	◯	◯	◯	◯
**1.3.6) Implementing the CYCLE protocol presented challenges to providing equitable service for all patients (i.e., patients not enrolled in CYCLE).**	◯	◯	◯	◯	◯	◯	◯	◯
**1.3.7) Having a CYCLE champion (e.g., PT and/or RN or study investigator) in the unit would be important to implementing the CYCLE protocol in our ICU.**	◯	◯	◯	◯	◯	◯	◯	◯

Champion - someone who supports and advocates for the CYCLE study and its participants.

1.4. Your role in CYCLE

Please choose the appropriate response for each item:

	Strongly disagree	Moderately disagree	Somewhat disagree	Neutral (neither agree nor disagree)	Somewhat agree	Moderately agree	Strongly agree	Not applicable
**1.4.1) When a patient was enrolled in CYCLE, ensuring they received their intervention or outcomes assessment was a high priority.**	◯	◯	◯	◯	◯	◯	◯	◯
**1.4.2) I enjoyed being part of the CYCLE study.**	◯	◯	◯	◯	◯	◯	◯	◯
**1.4.3) If the result of the CYCLE study was positive, I would consider cycling as a potential intervention to use with my patients.**	◯	◯	◯	◯	◯	◯	◯	◯

1.5 Is there anything not captured in the questions above that you would like to tell us about rehabilitation practice and research?

Please write your answer here:

2.1. Knowledge and beliefs about cycling

Please choose the appropriate response for each item:

	Strongly disagree	Moderately disagree	Somewhat disagree	Neutral (neither agree nor disagree)	Somewhat agree	Moderately agree	Strongly agree	Not applicable
**2.1.1) I am aware of the evidence for cycling with critically ill patients.**	◯	◯	◯	◯	◯	◯	◯	◯
**2.1.2) I believe that starting cycling early in ICU will improve patient outcomes at, or immediately post, ICU discharge.**	◯	◯	◯	◯	◯	◯	◯	◯
**2.1.3) I believe that starting cycling early in ICU will improve patient outcomes at hospital discharge.**	◯	◯	◯	◯	◯	◯	◯	◯

2.2. Implementing cycling

Please choose the appropriate response for each item:

	Strongly disagree	Moderately disagree	Somewhat disagree	Neutral (neither agree nor disagree)	Somewhat agree	Moderately agree	Strongly agree	Not applicable
**2.2.1) The bike was easily accessible.**	◯	◯	◯	◯	◯	◯	◯	◯
**2.2.2) The tablet worked reliably.**	◯	◯	◯	◯	◯	◯	◯	◯
**2.2.3) I had the technical skills to use the bike.**	◯	◯	◯	◯	◯	◯	◯	◯
**2.2.4) I felt confident in my ability to cycle with critically ill patients.**	◯	◯	◯	◯	◯	◯	◯	◯
**2.2.5) I could collect data (e.g., vital signs, cycling information) during a therapy session.**	◯	◯	◯	◯	◯	◯	◯	◯
**2.2.6) I required assistance from another person to transcribe data on research forms during a therapy session.**	◯	◯	◯	◯	◯	◯	◯	◯

2.3. Cycling delivery

Please choose the appropriate response for each item:

	Strongly disagree	Moderately disagree	Somewhat disagree	Neutral (neither agree nor disagree)	Somewhat agree	Moderately agree	Strongly agree	Not applicable
**2.3.1) If we were fully staffed, we could consistently deliver cycling sessions to patients enrolled in CYCLE.**	○	○	○	○	○	○	○	○
**2.3.2) Our team's communication facilitated consistent delivery of cycling sessions with enrolled patients (e.g., communicating re: temporary cycling exemptions).**	○	○	○	○	○	○	○	○
**2.3.3) Our team's coordination facilitated consistent delivery of cycling sessions with enrolled patients (e.g., scheduling cycling sessions).**	○	○	○	○	○	○	○	○
**2.3.4) When patients were enrolled in CYCLE, I felt an ethical responsibility to deliver the cycling intervention on all eligible days.**	○	○	○	○	○	○	○	○
**2.3.5) I would not cycle with a study patient if I believed other rehabilitation activities were a higher priority for them.**	○	○	○	○	○	○	○	○

2.4. Your feelings about cycling

Please choose the appropriate response for each item:

	Strongly disagree	Moderately disagree	Somewhat disagree	Neutral (neither agree nor disagree)	Somewhat agree	Moderately agree	Strongly agree	Not applicable
**2.4.1) I felt overwhelmed by cycling with critically ill patients.**	○	○	○	○	○	○	○	○
**2.4.2) I felt overwhelmed by the bike set-up and takedown.**	○	○	○	○	○	○	○	○
**2.4.3) I felt overwhelmed by the time required to conduct cycling sessions.**	○	○	○	○	○	○	○	○
**2.4.4) I had anxiety related to cycling with critically ill patients.**	○	○	○	○	○	○	○	○
**2.4.5) If I had a safety event during a cycling session, I would be reluctant to cycle again in the future.**	○	○	○	○	○	○	○	○

2.5. At this time, the results for the effectiveness of cycling are still unknown. However, if cycling improved outcomes and if it became recommended standard practice:

(please note that in some jurisdictions, both PT and OT are involved with early rehabilitation in the ICU)

Please choose the appropriate response for each item:

	Strongly disagree	Moderately disagree	Somewhat disagree	Neutral (neither agree nor disagree)	Somewhat agree	Moderately agree	Strongly agree	Not applicable
**2.5.1) ICU PT's should be responsible for implementing this intervention.**	○	○	○	○	○	○	○	○
**2.5.2) ICU OT's should be responsible for implementing this intervention.**	○	○	○	○	○	○	○	○

2.6. Do you believe another profession should be responsible for implementing cycling if it became standard of care?

Please choose **only one** of the following:

○ Yes

○ No

Please specify

Only answer this question if the following conditions are met:
Answer was 'Yes' at question '33 [C37]' (2.6. Do you believe another profession should be responsible for implementing cycling if it became standard of care?)

Please write your answer here:

2.7. Importance of leading cycling sessions

Please choose the appropriate response for each item:

	Not at all important	Of little importance	Of average importance	Neutral	Important	Very important	Extremely important	Not applicable
**How important is it for you to lead cycling sessions with patients enrolled in CYCLE?**	○	○	○	○	○	○	○	○

2.8. Ideally, if your site could receive additional resources to support your participation in CYCLE, how would you allocate these resources?

Please note that sites' allocation of research funds may be pre-specified and not able to change

❗ All your answers must be different and you must rank in order.

Please number each box in order of preference from 1 to 5

[ ] Provide backfill for therapists when a patient is enrolled on study

[ ] Provide ICU therapists additional compensation to cycle outside of regular working hours

[ ] Provide research personnel to deliver cycling sessions (also see question 2.10)

[ ] Provide a contribution to rehabilitation education fund (e.g., attendance at courses, invite speakers, accessing teleconferences and webinars)

[ ] Provide a contribution to rehabilitation equipment fund

2.9. Are there other ways that additional resources may be helpful?

Please choose **only one** of the following:

- ◯ Yes
- ◯ No

Please specify

Only answer this question if the following conditions are met:

Answer was 'Yes' at question '37 [C33]' (2.9. Are there other ways that additional resources may be helpful?)

Please write your answer here:

2.10. Enrollment is critical to study success. One way of helping improve enrollment is to offer help to study sites for **in-bed cycling**. In some jurisdictions both PT and OT are involved with early rehabilitation in the ICU. Please rate your comfort with the following people conducting in-bed cycling:

Please note that these people would be trained to use the bike and implement the study protocol.

Please choose the appropriate response for each item:

	Very uncomfortable	Moderately uncomfortable	Somewhat uncomfortable	Neutral (neither comfortable nor uncomfortable)	Somewhat comfortable	Moderately comfortable	Very comfortable
**2.10.1) ICU PT from study site**	◯	◯	◯	◯	◯	◯	◯
**2.10.2) Research PT with an ICU clinical background**	◯	◯	◯	◯	◯	◯	◯
**2.10.3) Research PT with no ICU clinical background**	◯	◯	◯	◯	◯	◯	◯
**2.10.4) Research PT with no clinical background at all**	◯	◯	◯	◯	◯	◯	◯
**2.10.5) PT student**	◯	◯	◯	◯	◯	◯	◯
**2.10.6) ICU OT from study site not currently participating in CYCLE**	◯	◯	◯	◯	◯	◯	◯
**2.10.7) Research OT with an ICU clinical background**	◯	◯	◯	◯	◯	◯	◯
**2.10.8) Research OT with no ICU clinical background**	◯	◯	◯	◯	◯	◯	◯
**2.10.9) Research OT with no clinical background at all**	◯	◯	◯	◯	◯	◯	◯
**2.10.10) OT student**	◯	◯	◯	◯	◯	◯	◯
**2.10.11) Research personnel (e.g., RT, RN) with an ICU clinical background**	◯	◯	◯	◯	◯	◯	◯
**2.10.12) Research personnel (e.g., RT, RN) with no ICU clinical background**	◯	◯	◯	◯	◯	◯	◯
**2.10.13) Research personnel (e.g., RT, RN) with no clinical background at all**	◯	◯	◯	◯	◯	◯	◯

2.11. Is there anyone else that you might consider delegating cycling to for the study?

Please choose **only one** of the following:

◯ Yes

◯ No

Please specify

Only answer this question if the following conditions are met:
Answer was 'Yes' at question '40 [C32]' (2.11. Is there anyone else that you might consider delegating cycling to for the study?)

Please write your answer here:

2.12. If we chose to delegate cycling, this could occur at different times during the course of the patient's stay. Please rate your comfort with delegating cycling at the following times:

Please note that people would be trained to use the bike and implement the study protocol.

Please choose the appropriate response for each item:

	Very uncomfortable	Moderately uncomfortable	Somewhat uncomfortable	Neutral (neither comfortable nor uncomfortable)	Somewhat comfortable	Moderately comfortable	Very comfortable
**2.12.1) Initial cycling session**	◯	◯	◯	◯	◯	◯	◯
**2.12.2) After a few cycling sessions**	◯	◯	◯	◯	◯	◯	◯

2.13. Would you be comfortable with delegating cycling at another time(s)?

Please choose **only one** of the following:

◯ Yes
◯ No

Please specify

Only answer this question if the following conditions are met:
Answer was 'Yes' at question '43 [C39]' (2.13. Would you be comfortable with delegating cycling at another time(s)?)

Please write your answer here:

2.14. With your site's current rehabilitation therapy staffing, how many CYCLE patients can you accommodate at a time?

2.15. If a cycling patient is discharged from ICU for less than 72 hours, would you be agreeable to enroll another patient in CYCLE?

Please choose **only one** of the following:

- ◯ Yes
- ◯ No

CYCLE patients who return to ICU within 72 hours and are still within the 28-day study period would resume the cycling intervention as per pre-ICU discharge.

2.16. Is there anything not captured in the questions above that you would like to tell us about your experiences with in-bed cycling for the CYCLE study?

Please write your answer here:

3.1. Knowledge of and practice with physical performance outcome measures (e.g., PFIT, 30-second sit-to-stand)

Please choose the appropriate response for each item:

	Strongly disagree	Moderately disagree	Somewhat disagree	Neutral (neither agree nor disagree)	Somewhat agree	Moderately agree	Strongly agree	Not applicable
**3.1.1) Conducting outcome measures with patients is part of my professional role.**	◯	◯	◯	◯	◯	◯	◯	◯
**3.1.2) Outcome measures reflect important differences in patients' physical function.**	◯	◯	◯	◯	◯	◯	◯	◯
**3.1.3) I routinely conduct outcome measures with critically ill patients.**	◯	◯	◯	◯	◯	◯	◯	◯
**3.1.4) I understand why we used the outcome measures in CYCLE.**	◯	◯	◯	◯	◯	◯	◯	◯
**3.1.5) The outcome measures used in CYCLE can be incorporated as part of routine therapy in the ICU.**	◯	◯	◯	◯	◯	◯	◯	◯

3.2. Implementing CYCLE outcome measures

Please choose the appropriate response for each item:

	Strongly disagree	Moderately disagree	Somewhat disagree	Neutral (neither agree nor disagree)	Somewhat agree	Moderately agree	Strongly agree	Not applicable
**3.2.1) I had the technical skills to conduct the outcome measures used in CYCLE.**	◯	◯	◯	◯	◯	◯	◯	◯
**3.2.2) I felt confident in my ability to collect the outcome measures used in CYCLE.**	◯	◯	◯	◯	◯	◯	◯	◯
**3.2.3) I could collect data (e.g., count steps) during outcome measure assessments.**	◯	◯	◯	◯	◯	◯	◯	◯
**3.2.4) I required assistance from another person to transcribe data on research forms during outcome measure assessments.**	◯	◯	◯	◯	◯	◯	◯	◯
**3.2.5) The equipment needed for the CYCLE outcome measures was easily accessible (e.g., stopwatch, trundle wheel, etc.).**	◯	◯	◯	◯	◯	◯	◯	◯

3.3. Outcome measure ascertainment

Please choose the appropriate response for each item:

	Strongly disagree	Moderately disagree	Somewhat disagree	Neutral (neither agree nor disagree)	Somewhat agree	Moderately agree	Strongly agree	Not applicable
**3.3.1) If we were fully staffed, we could consistently collect outcome measures for patients enrolled in CYCLE.**	◯	◯	◯	◯	◯	◯	◯	◯
**3.3.2) It was feasible to conduct outcomes assessments and cycling sessions with patients on the same day.**	◯	◯	◯	◯	◯	◯	◯	◯
**3.3.4) Patients' functional capacity or fatigue limited conduct of cycling and outcome measures on the same day.**	◯	◯	◯	◯	◯	◯	◯	◯
**3.3.5) If I had limited time or a patient was tired, I knew which outcome measures to prioritize collecting.**	◯	◯	◯	◯	◯	◯	◯	◯
**3.3.6) Our team's communication facilitated consistent collection of CYCLE outcome measures with eligible patients (e.g., communicating with research coordinator once patient passed cognitive screen).**	◯	◯	◯	◯	◯	◯	◯	◯
**3.3.7) Our team's coordination facilitated consistent collection of CYCLE outcome measures with eligible patients (e.g., scheduling outcomes assessments).**	◯	◯	◯	◯	◯	◯	◯	◯

3.4. Your feelings about outcome measures

Please choose the appropriate response for each item:

	Strongly disagree	Moderately disagree	Somewhat disagree	Neutral (neither agree nor disagree)	Somewhat agree	Moderately agree	Strongly agree	Not applicable
**3.4.1) I felt overwhelmed by collecting outcome measures with critically ill patients.**	◯	◯	◯	◯	◯	◯	◯	◯
**3.4.2) I felt overwhelmed by the equipment set-up and takedown needed for the outcome measures.**	◯	◯	◯	◯	◯	◯	◯	◯
**3.4.3) I felt overwhelmed by the time required to collect outcome measures.**	◯	◯	◯	◯	◯	◯	◯	◯
**3.4.4) I felt overwhelmed by the number of outcome measures to collect.**	◯	◯	◯	◯	◯	◯	◯	◯
**3.4.5) I felt anxious when I knew I had to conduct outcome measures with a CYCLE patient.**	◯	◯	◯	◯	◯	◯	◯	◯

3.5. Outcome measure intentions

Please choose the appropriate response for each item:

	Strongly disagree	Moderately disagree	Somewhat disagree	Neutral (neither agree nor disagree)	Somewhat agree	Moderately agree	Strongly agree	Not applicable
**3.5.1) I intend to use the outcome measures from CYCLE as part of usual care.**	◯	◯	◯	◯	◯	◯	◯	◯

3.6. Please describe how your site coordinated blinded outcomes assessments.

Please write your answer here:

3.7. Is there anything not captured in the questions above that you would like to tell us about your experiences with outcome measures for the CYCLE study?

Please write your answer here:

4.1. From your perspective, what were the three **easiest** parts of the CYCLE study to implement?

E.g., collecting outcome measures, coordinating sessions, recruitment and enrolment, cycling set-up and takedown, etc.

4.2. From your perspective, what were the three **most difficult** parts of the CYCLE study to implement?

E.g., collecting outcome measures, coordinating sessions, recruitment and enrolment, cycling set-up and takedown, etc.

4.3. Do you have any tips to share with other therapists or study coordinators to make the study more successful?

Please write your answer here:

4.4. Do you have any suggestions to improve the next phase of the CYCLE study?

Please write your answer here:

4.5. Is there anything else you would like us to know?

Please write your answer here:

Thank you for taking this survey. Your answers are a valuable part of this research.

09-20-2018 – 09:23

Submit your survey.
Thank you for completing this survey.
